# First person – Yong Chen

**DOI:** 10.1242/dmm.043430

**Published:** 2019-12-09

## Abstract

First Person is a series of interviews with the first authors of a selection of papers published in Disease Models & Mechanisms (DMM), helping early-career researchers promote themselves alongside their papers. Yong Chen is first author on ‘Formula feeding and immature gut microcirculation promote intestinal hypoxia, leading to necrotizing enterocolitis’, published in DMM. Yong conducted the research described in this article while a research fellow in Agostino Pierro's lab at The Hospital for Sick Children, Toronto, Canada. He is now a staff physician in the Department of Pediatric Surgery at KK Women's and Children's Hospital, as well as adjunct assistant professor at Duke-NUS Medical School, Singapore, investigating the aetiology, treatment and prevention of necrotizing enterocolitis.


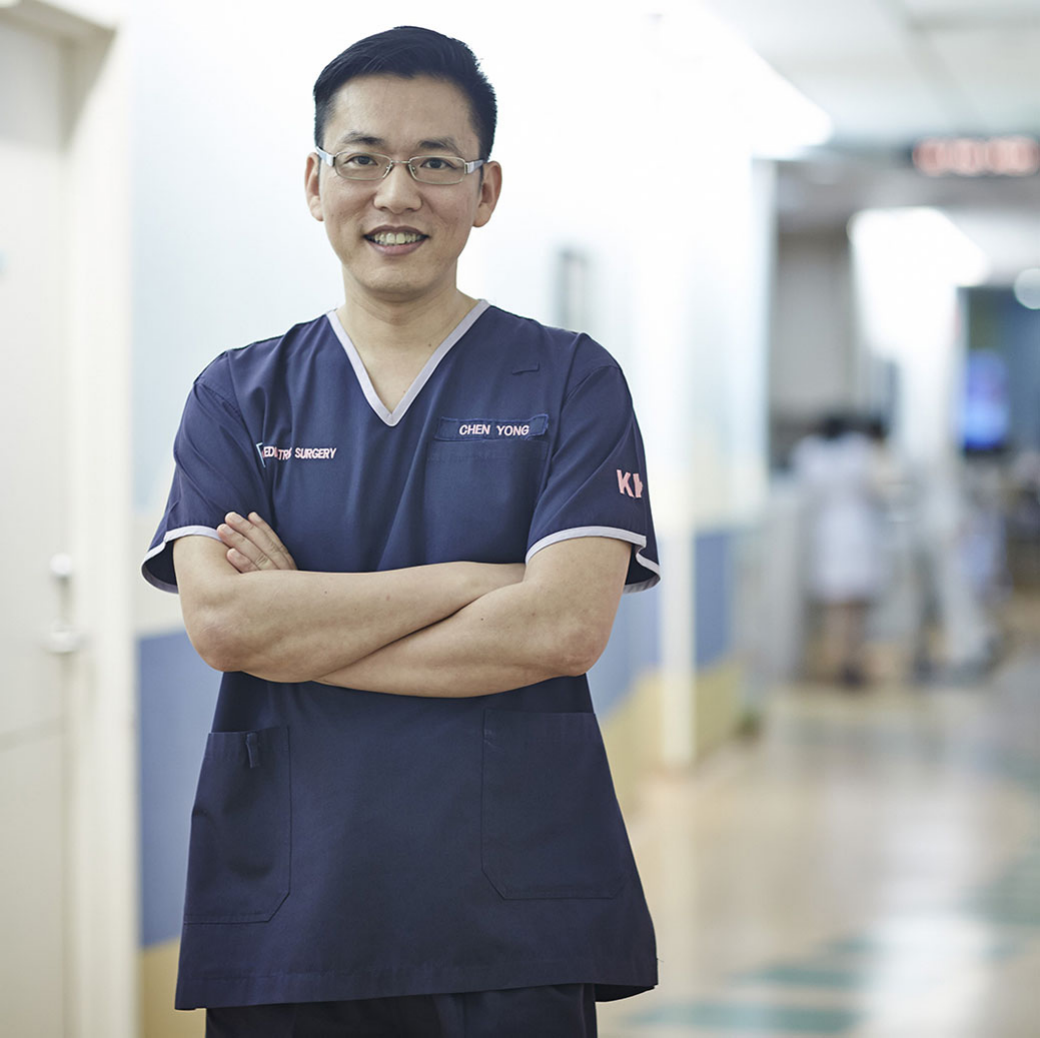


**Yong Chen**

**How would you explain the main findings of your paper to non-scientific family and friends?**

Necrotizing enterocolitis (NEC) is a severe disease that predominantly affects premature infants, causing bowel death. It mainly occurs after an infant is fed large quantities of milk, but the cause is not clear. In a mouse model, we found that the bowel needs oxygen for digestion, but the immature gut has a poor blood supply and cannot provide sufficient oxygen after feeding. This shortage of oxygen then causes damage to the bowel, resulting in NEC. We found that the premature gut is unable to produce enough arginine, an important amino acid that increases bowel blood flow after feeding. Interestingly, adding arginine to milk can prevent NEC in mouse pups.

**What are the potential implications of these results for your field of research?**

The incidence of NEC remains unchanged over the past few decades due to poor understanding of its aetiology. The onset of NEC is likely contributed to by multi-factors such as prematurity, enteral feeding, inflammation and hypoxia/ischaemia. However, it remains unclear how these risk factors work together to trigger NEC. We have created a mouse model that allows us to directly visualize the microcirculation of the intestine *in vivo*. Through this model, we revealed that insufficient bowel blood supply in premature gut and increased oxygen demand after feeding synergistically leads to intestinal hypoxia and subsequent NEC in mice. If the disease model is translatable to a human infant, a balanced feeding quantity meeting the early preterm infant's gut blood flow would reduce the burden of NEC. Furthermore, supplementation of arginine would increase intestinal vessel dilation and prevent tissue hypoxia and NEC.

“The main advantage of our model system is the high-resolution, real-time monitoring of the microcirculation in live animals, which allows us to investigate the haemodynamics of the bowel after feeding.”

**What are the main advantages and drawbacks of the model system you have used as it relates to the disease you are investigating?**

In this study, we have created a model system to investigate intestinal microcirculation *in vivo* using *Rosa^mT/mG/+^;Tie2-Cre* transgenic mice under two-photon microscopy. The main advantage of our model system is the high-resolution, real-time monitoring of the microcirculation in live animals, which allows us to investigate the haemodynamics of the bowel after feeding. The specific GFP expression in endothelial cells of transgenic mice also allows us to isolate intestinal endothelial cells and investigate the molecular pathways that contribute to insufficient blood flow in the premature gut. The main drawback is that the imaging system is based on transgenic mice and not applicable to humans. Other methods, such as ultrasound Doppler analysis, are needed to reveal the role of intestinal blood flow and enteral feeding in the pathogenesis of NEC in human infants.

**What has surprised you the most while conducting your research?**

We have analysed the whole-genome expression profile in intestinal endothelial cells of early and late neonatal mice. I surprisingly found that out of 25,000 detected transcripts, the arginine biosynthesis pathway was the most significantly upregulated pathway in late neonatal mice. Arginine is a precursor of nitric oxide, an important vasodilator. This finding suggests that compromised arginine production in the premature gut contributes to insufficient blood supply and increases the risk of NEC in infants.

**Figure:**
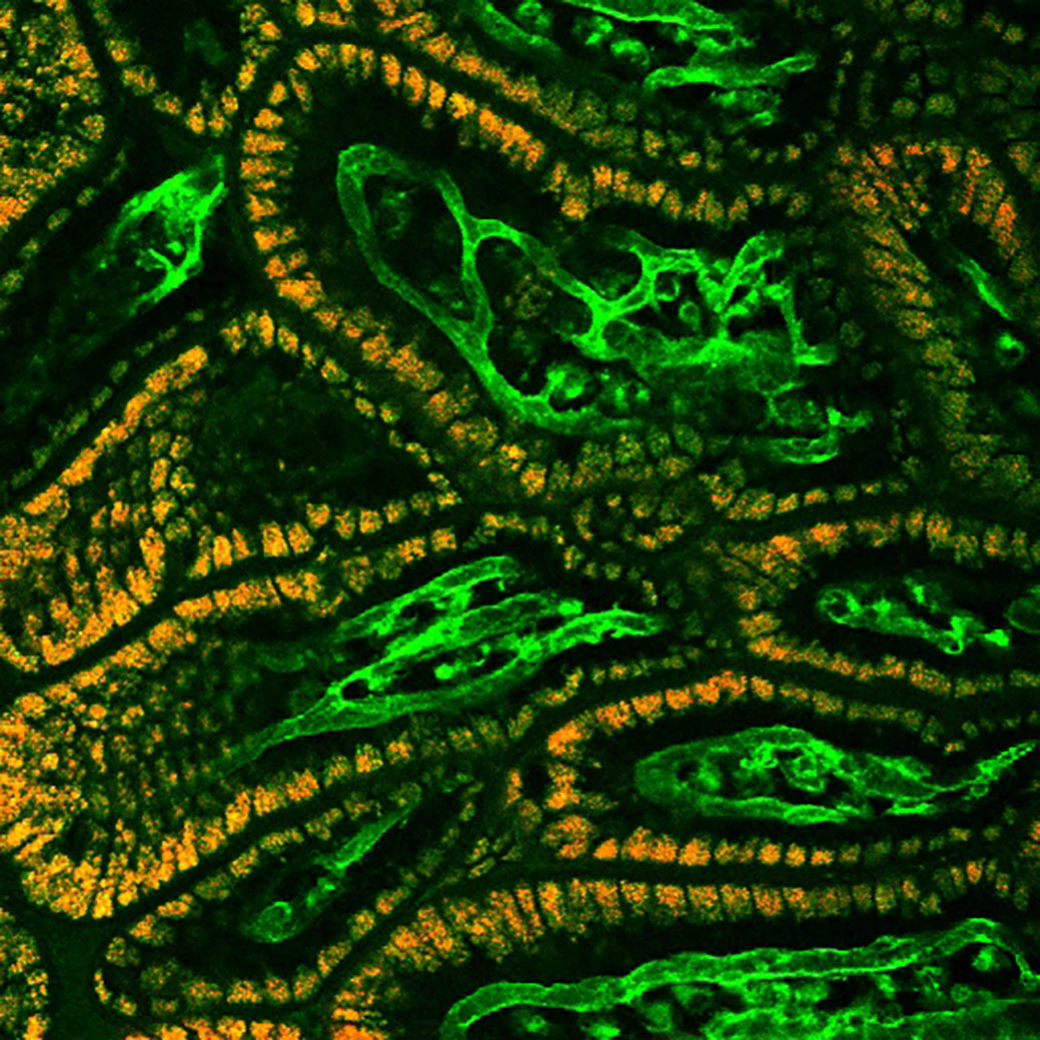
**The expression of tdTomato (red) in epithelial and GFP (green) in endothelial cells in intestinal villi of *Rosa^mT/mG/+^;Tie2-Cre* transgenic mice.**

**Describe what you think is the most significant challenge impacting your research at this time and how will this be addressed over the next 10 years?**

The most significant challenge at this time is to verify the findings of animal studies in humans and reveal the pathogenesis of NEC in premature infants. Understanding the aetiology of NEC could help us to develop a strategy to prevent and treat this devastating disease, which has a mortality rate of around 30%. I believe that in the next 10 years, the findings and treatment strategies developed from animal studies (such as regulation of gut microbiota, adjusting feeding based on intestinal microcirculation) will significantly reduce the incidence and mortality of NEC.

“It is important not to give up easily but not to stick to a wrong direction for too long.”

**What changes do you think could improve the professional lives of early-career scientists?**

Being a scientist, we have to face many challenges and failures during research work. It is important not to give up easily but not to stick to a wrong direction for too long. Communication and sharing ideas between researchers with different specialties can help us to figure out the correct way, leading to success of the project.

**What's next for you?**

I will continue to research NEC and try to verify the findings of our animal work in clinical study. Eventually, we aim to develop a feeding regime that will decrease oxygen demand and increase intestinal blood supply to prevent NEC.
